# Higher Efficiency of Percutaneous Microwave (MWA) Than Radiofrequency Ablation (RFA) in Achieving Complete Response in Cirrhotic Patients with Early Hepatocellular Carcinoma

**DOI:** 10.3390/curroncol28020101

**Published:** 2021-02-25

**Authors:** Silvia Gaia, Michela Ciruolo, Davide Giuseppe Ribaldone, Emanuela Rolle, Enrica Migliore, Elena Mosso, Simone Vola, Alessandra Risso, Sharmila Fagoonee, Giorgio Maria Saracco, Patrizia Carucci

**Affiliations:** 1 Gastroenterology and Hepatology Unit, Città della Salute e della Scienza University-Hospital, 10126 Turin, Italy; michela.ciruolo@edu.unito.it (M.C.); emanurolle@inwind.it (E.R.); elena.mosso@aslbi.piemonte.it (E.M.); simonevola@libero.it (S.V.); alrisso@cittadellasalute.to.it (A.R.); giorgiomaria.saracco@unito.it (G.M.S.); pcarucci@cittadellasalute.to.it (P.C.); 2 Department of Medical Sciences, University of Turin, 10126 Turin, Italy; davidegiuseppe.ribaldone@unito.it; 3 Unit of Cancer Epidemiology, Città della Salute e della Scienza University-Hospital and Center for Cancer Prevention (CPO), 10126 Turin, Italy; emigliore@cittadellasalute.to.it; 4 Institute for Biostructure and Bioimaging (CNR) at Molecular Biotechnology Center, 10126 Turin, Italy; sharmila.fagoonee@unito.it

**Keywords:** microwave ablation, hepatocellular carcinoma, radiofrequency ablation, locoregional therapy, necrosis, percutaneous techniques, survival

## Abstract

Background: Contrasting data are available in the literature regarding the superiority of percutaneous microwave ablation (MWA) or radiofrequency ablation (RFA) in very early or early (BCLA 0 or A) hepatocellular carcinoma (HCC). Aims: The primary outcome was to compare the efficacy of RFA and MWA in achieving complete response in cirrhotic patients with early and very early HCC. The secondary outcomes were to evaluate the overall survival and the recurrence rate. Methods: A retrospective, observational, single-center study was performed. Inclusion criteria were liver cirrhosis, new diagnosis of a single node of HCC measuring a maximum of 50 mm or up to three nodules with diameter up to 35 mm, treatment with RFA or MWA. Radiological response was evaluated with multiphasic contrast-enhanced Computed Tomography or Magnetic Resonance Imaging at 5–7 weeks after thermal ablation. Complete response was defined when no vital tissue was detected after treatment. Results: Overall, 251 HCC patients were included in this study; 81 patients were treated with MWA and 170 with RFA. The complete response rate was similar in MWA and RFA groups (out of 331 nodules, 87.5% (91/104) were treated with MWA and 84.2% (186/221) were treated with RFA, *p* = 0.504). Interestingly, a subanalysis demonstrated that for 21–35 mm nodules, the probability to achieve a complete response using MWA was almost 5 times higher than for RFA (OR = 4.88, 95% CI 1.37–17.31, *p* = 0.014). Moreover, recurrence rate in 21–35 mm nodules was higher with RFA with respect to MWA (31.9% versus 13.5%, *p* = 0.019). Overall survival was 80.4% (45/56) when treated with MWA and 62.2% (56/90) when treated with RFA (*p* = 0.027). No significant difference was observed between MWA and RFA treatment in the 15–20 mm nodules group. Conclusion: This study showed that MWA is more efficient than RFA in achieving complete response in HCC nodules with 21 to 35 mm diameter.

## 1. Introduction

Hepatocellular carcinoma (HCC) represents about 90% of primary liver cancers. Currently, HCC is the seventh most common cancer and the second for mortality in the world, and its incidence and mortality rates are expected to increase in the upcoming decades [1,2]. HCC therapy is complex and involves several strategies depending on the stage (resection, percutaneous techniques, liver transplantation, chemoembolization, radioembolization, radiotherapy, target therapy), and multiple options may be available to the same patient [3,4]. According to the Barcelona Clinic Liver Cancer (BCLC) staging system, radiofrequency ablation (RFA) and microwave ablation (MWA) are widely employed treatments for very early (BCLC 0) and early (BCLC A) HCC [5,6]. The assessment of response to locoregional HCC therapy is evaluated with multiphasic contrast-enhanced Computed Tomography (CT) or Magnetic Resonance Imaging (MRI), relying on mRECIST criteria [7].

Contrasting data are available on the efficiency of RFA and MWA. In clinical practice, HCC nodes with a diameter from 15 to 35 cm are treated indifferently with RFA or MWA, depending on the choice of the clinician. Several studies comparing RFA with MWA indicated a similar efficacy between the two percutaneous techniques, while one study showed possible superiority of MWA in HCC with larger nodules [8].

RFA is based on generation of a current (370 to 505 kHz) through an electrode tip inserted into the tumoral node that induces local heat (55 to 95 °C) and causes necrosis. The heat propagates in a centrifugal direction and the temperature decreases. This is the cause of the decrease of its efficacy in HCC nodes larger than 20 mm. MWA is a thermal technique that creates an electromagnetic field around a monopolar electrode, inducing homogeneous heating and necrosis. MWA offers several advantages over the other forms of thermal ablation such as: inducing larger volumes of necrosis; reaching faster ablation rates; increasing the sphericity of the necrosis area due to development; and application of high-powered antennas and new powerful generators [9,10,11].

The aim of the study is to compare the efficacy of RFA and MWA in achieving complete response in cirrhotic patients with early and very early HCC. Moreover, the overall survival, the recurrence rate in patients treated with RFA and MWA, and the variables that may influence the outcomes were evaluated.

## 2. Materials and Methods

### 2.1. Patients

This retrospective observational study included all consecutive patients with cirrhosis and first diagnosis of HCC treated with percutaneous thermal ablation by RFA and MWA at the Gastroenterology and Hepatology Unit, “Città della Salute e della Scienza” University-Hospital, Turin, Italy, from January 2013 to May 2019. Diagnosis of HCC and allocation treatment followed BCLC and EASL guidelines [10,12]. 

Inclusion criteria were the following: patients with liver cirrhosis (Child Pugh score A or B), new diagnosis of a single node of HCC measuring a maximum of 50 mm or up to three nodules with diameter up to 35 mm, treated with percutaneous ultrasound (US)-guided RFA or MWA. The choice between RFA or MWA depended on local availability and on the preference of the operator. All the procedures were performed by 3 highly experienced hepatologists with more than 6 years of US-guided thermal ablation activity. Other treatments or combination therapies were excluded. Demographic, clinical, and radiological data were prospectively collected at the time of intervention, and patients were subsequently followed up and staged at our hospital. 

Data regarding aetiology of cirrhosis, hepatic function (Child Pugh-score), age, sex, body mass index (BMI), alcohol and smoking habits, number of lesions and their radiological characteristics were collected. Specifically, HCC nodules were examined for maximum diameter of lesion in cases of a single tumor, sum of sizes in cases of multiple lesions (sum of diameters-SOD), number of lesions, complex position (perivascular, subdiaphragmatic, pericholecystic, subcapsular lesions) and ultrasound visibility (poor or good visibility).

### 2.2. HCC Nodules: Evaluation of the Tumor Response

Only well-delineated, arterially enhancing lesions that could be measured and selected as target lesions for EASL criteria [10] at pretherapy multiphasic contrast-enhanced CT or MRI were included in the study. Radiological response was evaluated following EASL criteria with multiphasic contrast-enhanced CT or MRI at 5–7 weeks after first treatment. Further, patients were followed by specialist abdominal US with or without contrast enhancement every 4 months, and CT or MRI yearly, following EASL guidelines [10]. End of follow-up was considered at study end (15 May 2019) or when death occurred or upon orthotopic liver transplantation. All patients signed an informed consent for the procedure and for data collection in respect of their privacy following the Ethical Committee approval (Italian Legislative Decree No. 196 dated 30 June 2003). Complete response is defined as the disappearance of any intratumoral arterial enhancement; residual viable tumor tissue is defined as the arterially enhancing tissue within the treated HCC, and it is measured to assess treatment response. Recurrence is defined, after a complete response, as the presence of vital tumor occurring locally. 

### 2.3. Statistical Analysis

Baseline characteristics of all patients included in the analysis are summarized using median and interquartile range (IQR), and percentages and frequencies (n, %). Between-group differences in sociodemographic and clinical characteristics at HCC diagnosis were evaluated by the Mann–Whitney U test for continuous variables and the Chi-square test or Fisher’s exact test for categorical variables. Complete radiological response in the subpopulation of nodules with a diameter from 15 to 35 mm was evaluated using a logistic regression model, overall and between diameter groups (15–20 mm vs. 21–35 mm). Crude and adjusted Odds Ratios (ORs) and their 95% confidence intervals (95% CI) were reported. Multivariable models included all factors known to have a predictive role on response (nodules position and visibility, Child Pugh class, number of lesions, and diameter of lesions).

To describe the timing of recurrence, the cumulative incidence of recurrence was estimated. Statistical significance (*p* < 0.05) of differences in the cumulative incidence of recurrence between groups (i.e., by treatment, diameter group, position, visibility) was tested using the log-rank test for homogeneity. The observation period for time to recurrence started on the day of thermoablation treatment until the date of recurrence diagnosis (failures), or the last follow-up visit (censoring). Patients who underwent orthotopic liver transplantation were censored at the time of intervention. In this analysis, death was considered as a competing event. We estimated Subdistributional Hazard Ratios (SHR) in a semiparametric model according to Fine and Gray to evaluate possible predictors of recurrence and plotted the cumulative incidence function. The overall survival, estimated with the Kaplan–Meier method, was defined as the time from thermoablation to death by any cause; patients alive were censored on the date of the last follow-up. Orthotopic liver transplantation was considered a time-dependent variable in the model. Potential prognostic variables were evaluated as predictors of survival by the Cox proportional hazard model to estimate the crude and the multivariable-adjusted Hazard Ratios (HRs) with 95% Confidence Intervals (CIs) and to evaluate possible predictors of survival. The proportional hazard assumption was also verified by graphical checks and formal tests based on Schoenfeld residuals. The variables that a priori were considered to have clinical relevance, such as nodule position, diameter, visibility, and number of lesions present, were included in the multivariable model. The analysis was performed by Stata 15.1 software (StataCorp LP, College Station, TX, USA). 

## 3. Results

### 3.1. Patients

Overall, 251 HCC patients with 331 nodules were included in this study. Median age was 64 years old (IQR 56–73), with 87% in Child Pugh class A and 13% in Child Pugh class B at the moment of thermal ablation. A total of 81 patients were treated with MWA and 170 with RFA; the two groups of patients were homogeneous in terms of demographical and clinical features. However, the median time of follow-up was 2.48 years (IQR 1.32–3.81) and was significantly longer in patients treated with RFA compared with those treated with MWA: 2.94 (IQR 1.61–4.25) vs. 1.66 (0.86–3.20), *p* < 0.001. Patient details are described in Table 1.

Overall, the 331 HCC nodules included had a median of maximum diameter of 21 mm (IQR 16–28). At inclusion time, 75% (189) of cases had a single HCC nodule, 19% (48) had two nodules, and 5.6% (14) had 3 nodules; 106 nodules (32%) were treated with MWA and 225 (68%) with RFA. The nodules treated with MWA were significantly larger than those treated with RFA, with a median diameter of 29 mm (IQR 20–35 mm) vs. 20 mm (IQR 15–25 mm), respectively, *p* < 0.001. The median of maximum diameter and of SOD, in the case of multiple lesions for each patient, was 27 mm (IQR 20–35): 33 mm (IQR 27–45) in the MWA group and 25 mm (19–31) in the RFA group, respectively.

Features of all included nodules treated with RFA and MWA are reported in Table 2. 

### 3.2. Radiological Response and Recurrence

**Figure 1:**
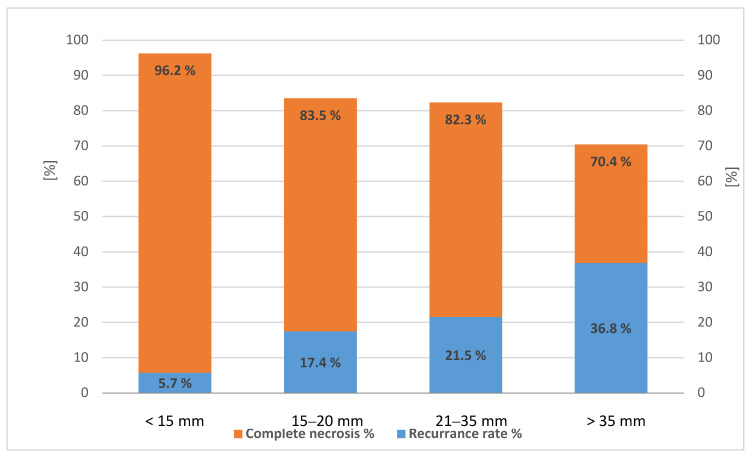
Rate of complete necrosis (left axis) and recurrence rate (right axis) in 331 hepatocellular carcinoma (HCC) nodules treated with any percutaneous thermal ablation, *p* < 0.05 (test for trend).

Complete radiological response was achieved in 83.7% (277/331) of nodules treated with any thermoablation therapy. The rate of complete response was dependent on nodule diameter, and the risk of recurrence was related to the dimension of the single nodule treated as shown in Figure 1.

The complete response rate was similar in MWA and RFA (87.5%, 91/104 of nodules treated with MWA and 84.1%, 186/221 of nodules treated with RFA, respectively, *p* = 0.504). Six patients were missing: four patients were lost to follow-up, and two patients underwent liver transplantation three and 20 days after ablation). The cumulative incidence of recurrence in the whole population was similar in the two groups of treatment. In fact, the cumulative incidence of recurrence at 6, 12 and 18 months was, respectively, 2.5%, 8.8% and 14% in the MWA group, and 1.8%, 8.9% and 14.4% in the RFA group (*p* > 0.05). At multivariable analysis, the cumulative incidence of recurrence was influenced by the maximum diameter of the HCC nodules (SHR 1.04, 95%CI 1.00–1.08, *p* = 0.042) and was not related to type of therapy and other nodule features such as position and US visibility. In case of recurrence, the tumor was thermal ablated again if possible or treated with other techniques such as trans arterial chemoembolization or stereotactic radiotherapy, if indicated, or patient underwent liver transplantation (data not shown). 

### 3.3. Sub-Analysis on Nodules with Diameter between 15 and 35 mm

Considering that very big nodules (larger than 35 mm) were treated primarily with MWA and small nodules (inferior to 15 mm) with RFA, as shown in Table 2, in order to make the two groups of therapy more comparable, we performed a subanalysis on 250 HCC nodules with a diameter of 15 to 35 mm. A total of 78 nodules (31%) were treated with MWA and 172 (69%) with RFA. Nodules treated with MWA were significantly larger (median diameter of 25 mm (IQR 20–30 mm)) compared to those treated with RFA (mean diameter of 21 mm (IQR 18–25), *p* < 0.001).

### 3.4. Complete Response in Nodules with Diameter between 15 and 35 mm

**Figure 2:**
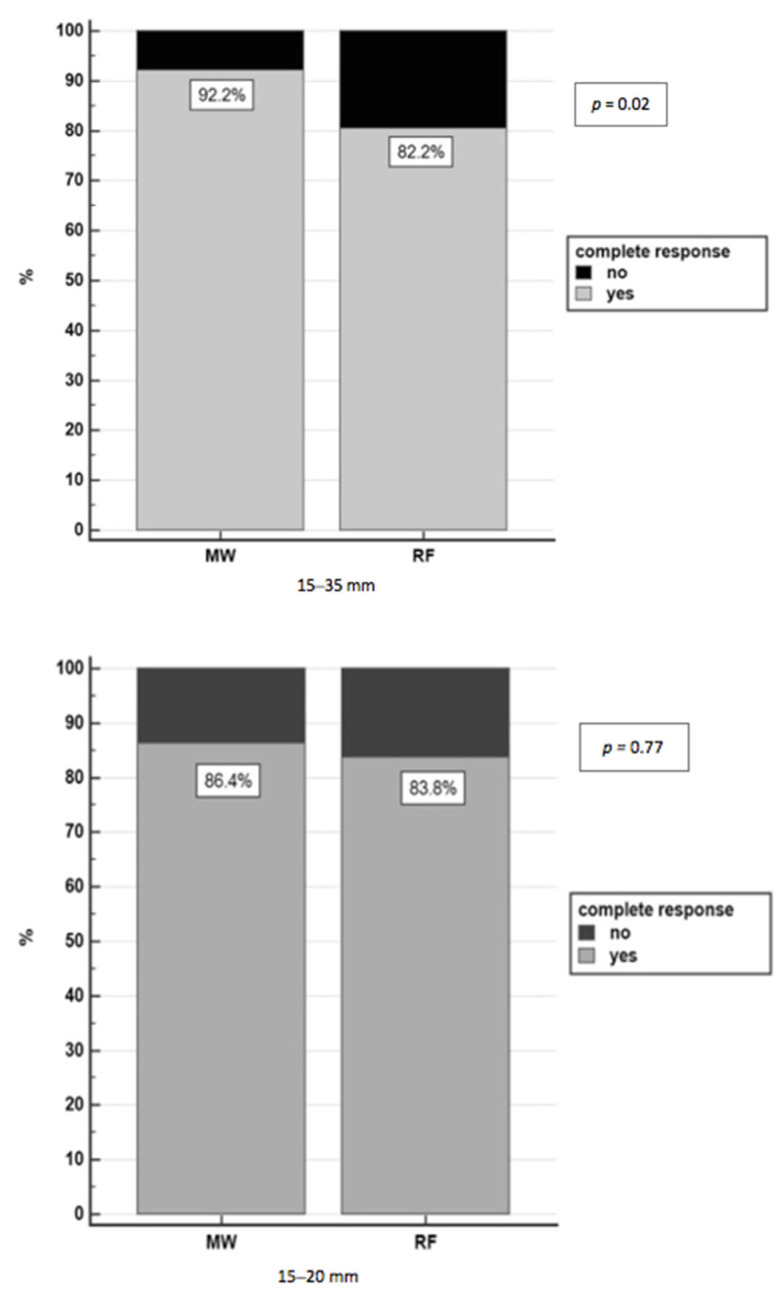

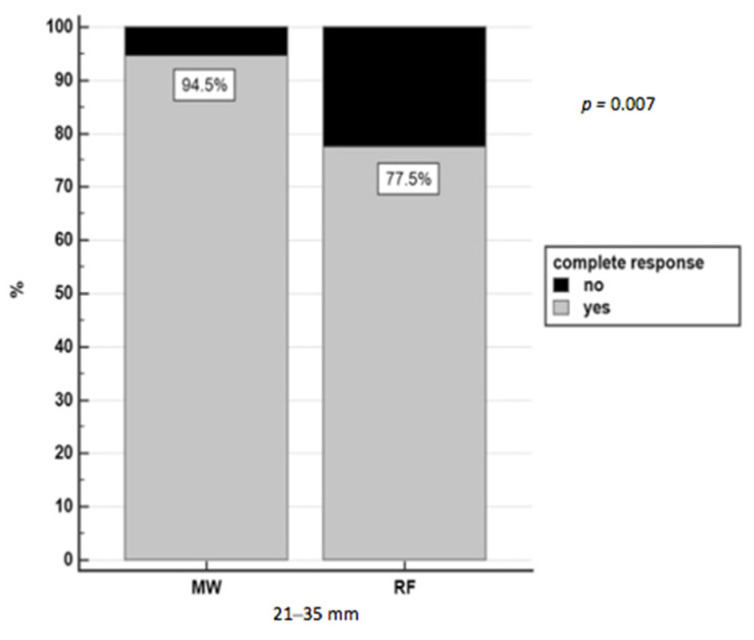
Complete response in nodules 15–35 mm treated with RFA and MWA.

Complete response was achieved with thermoablation in 84.1% (207/246) of nodules (in four cases the data were not available: three patients were lost to follow-up, and one patient underwent liver transplantation three days after ablation). MWA achieved a complete response in a higher rate of HCC patients with respect to RFA: 92.2% (71/77) of nodules vs. 80.5% (136/169), respectively, *p* = 0.02, and, in particular, in nodules with a diameter of 21–35 mm group (*p* = 0.007) (Figure 2).

Following the logistic model, 15–35 mm nodules treated with MWA had 3.26 times higher probability of achieving complete necrosis (95% CI 1.04–10.2, *p* = 0.042) than RFA, independently of the nodule position, visibility, maximum diameter, and Child Pugh class. Further analysis was performed on two subgroups of nodules based on diameter (15–20 mm and 21–35 mm), homogeneity in terms of type of therapy, number of lesions for each patient, complex position (perivascular, subdiaphragmatic, pericholecystic, subcapsular lesions), US visibility and infiltrative US pattern (*p* = 0.500) as shown in Supplementary Table S1. 

Taking into account the small 15–20 mm nodules, there was no difference in achieving complete response between MWA and RFA (OR = 1.37, 95%CI 0.36–5.19, *p* = 0.641), while for bigger 21–35 mm nodules, the probability to achieve a complete response using MWA was almost five times higher than RFA (OR = 4.88, 95% CI 1.37–17.31, *p* = 0.014).

### 3.5. Recurrence Rate in Nodules with Diameter between 15 and 35 mm

In the HCC nodules with a diameter of 15 to 35 mm, the mean recurrence rate after complete response was 22.2% (46/207): 14.1% (10/71) in nodules treated with MWA and 26.5% (36/136) in those treated with RFA (*p* = 0.04). For 15–20 mm nodules, the recurrence rate was similar in the MWA and RFA treatment groups (15.8%, 3/19 vs 20.9%, 14/67, respectively, *p* = 0.62), while for bigger 21–35 mm nodules, the recurrence rate was 13.5% (7/52) in nodules treated with MWA and 31.9% (22/69) in those treated with RFA (*p* = 0.019). At multivariate analysis, an increasing trend of recurrence risk was observed for 21–35 mm nodules treated with RFA (OR 2.57, 95% CI 0.95–6.94, *p* = 0.062) compared to 15–20 mm nodules, while the recurrence rate was independent of other HCC features such as type of therapy, position and US visibility.

### 3.6. Overall Survival 

**Figure 3:**
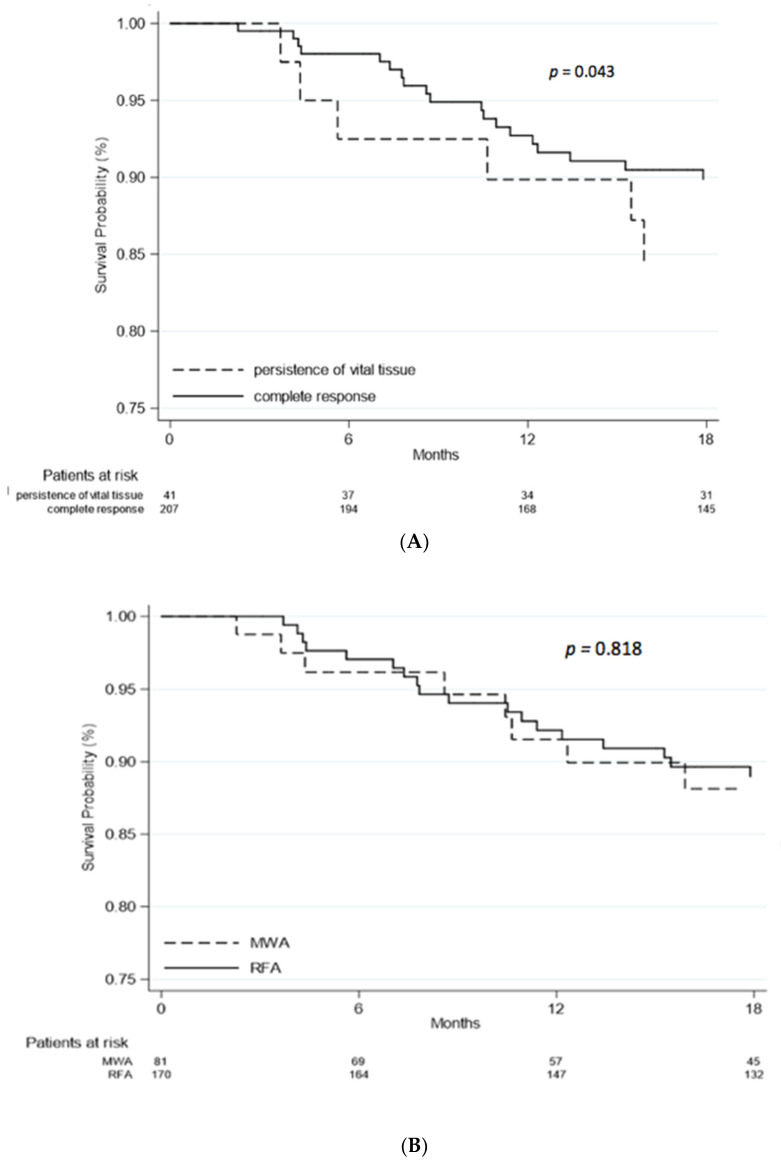
(**A**), Overall survival; (**B**), Overall survival by treatment. Demographic features of patients at baseline, alfa-fetoprotein value at diagnosis and type of therapy were not related to survival rate (*p* > 0.05). Patients who underwent liver transplantation after thermal ablation had a better survival rate compared to the others (HR = 0.19; 95%CI 0.06–0.57, *p* = 0.003).

Death occurred in 58 out of 251 (23%) patients. At multivariate analysis, overall survival was related to the Child Pugh A vs. B score (HR = 3.46, 95%CI 1.68–7.13, *p* = 0.001), and to the SOD (HR = 1.04, 95%CI 1.01–1.07, *p* = 0.005). Patients who achieved complete response after thermoablation showed increased overall survival compared to patients with persistence of vital tissue (*p* = 0.043, see Figure 3A). The mortality risk between patients treated with RFA (N = 15, 18.5%) and MWA (N = 43, 25.3%) was similar (HR = 0.97, 95%CI 0.50–1.89, *p* = 0.938). In Figure 3B, the overall survival probability of patients in the two groups of treatment is reported.

### 3.7. Overall Survival in Nodules with Diameter between 15 and 35 mm

Overall, in HCC nodules with a diameter of 15 to 35 mm, the mean overall survival was 73.5% (183/249) (in one case the data were not available): 83.3% (65/78) in nodules treated with MWA and 69% (118/171) in those treated with RFA (*p* = 0.02). In small 15–20 mm nodules, the overall survival was 90.9% (20/22) in nodules treated with MWA and 76.5% (62/81) in those treated with RFA (*p* = 0.23), while for bigger 21–35 mm nodules, the overall survival was 80.4% (45/56) in nodules treated with MWA and 62.2% (56/90) in those treated with RFA (*p* = 0.027). 

## 4. Discussion

In the present study, we compared the efficacy of percutaneous treatment with RFA and MWA as a curative purpose in patients with early and very early HCC. Complete necrosis of tumor tissue was achieved in 84% of nodules without significant differences between MWA and RFA when all HCC nodules up to 50 mm were considered. However, in a subgroup of nodules with a diameter of 21 to 35 mm, MWA was superior in achieving complete response compared to RFA (*p* = 0.007). Considering 15–35 mm HCC nodules, those treated with MWA had a higher probability of obtaining a complete necrosis than RFA, independently of the nodule position, visibility, and Child Pugh class. Recurrence rate was lower for MWA with respect to RFA when considering nodules with a diameter of 21 to 35 mm: the recurrence rate was 13.5% in nodules treated with MWA and 31.9% in those treated with RFA. Overall survival depended on the efficacy of treatment (complete response vs. persistent of vital tissue), liver function (Child Pugh A vs. B), and liver transplantation after treatment. Taking into account 21–35 mm nodules, the overall survival was 80.4% in nodules treated with MWA and 62.2% in those treated with RFA. Overall, these results show that MWA achieved superior complete response in nodules larger than 21 mm compared to RFA in HCC patients.

Several studies have compared modern MWA and RFA techniques with contrasting results. Qian et al. found no difference between MWA and RFA in long-term progression in nodules less than 3 cm in diameter [13]. Potretzke et al. found a 2-fold increased risk of long-term progression after RFA with respect to MWA: HCC greater than 3 cm in diameter showed the greatest difference in progression risk between MWA and RFA [14]. A recent study published by Bouda et al. found a higher efficacy of MWA compared to RFA ablation in 149 patients with naive HCC at very early or early stage in terms of local tumor progression, but they did not evaluate overall survival [15]. On the other hand, the study of McDevitt et al. showed no difference in the risk of primary failure or local progression between RFA and MWA in 136 patients. However, the authors did not perform a subanalysis in the two groups according to diameter measurement (15–20 mm and 21–35 mm) [16]. 

Considering reviews and meta-analyses, no differences in local recurrence for up to five years after treatment were found comparing MWA and RFA, and MWA is recommended only for lesions >3 cm [8,17,18,19,20]. A possible explanation for the higher complete response rate with MWA found in the 21–35 mm group in comparison with the 15–20 mm group in our study could be that MWA antenna can achieve continuous and faster heating, thus generating a larger area of ablation than RFA [21,22]

This study has some limitations, principally due to the retrospective design (despite the fact that demographic, clinical and radiological data were prospectively collected at the moment of intervention and periodically revised to avoid missing data), single-center study, lack of data about complications related to the procedures, and the nonrandomized treatment allocation. All consecutive 251 patients with cirrhosis and first diagnosis of HCC treated with thermal ablation at our department were included to avoid biases in selection. The choice for MWA or RFA was taken by the single operator based on personal experience, that is, small nodules were preferably treated with RFA while MWA was preferred for bigger or numerous nodules. RFA was performed by a multiple-hooked needle and was preferred in cases of unstable position into the liver. MWA was performed with a single-hook needle and conferred a bigger necrotic area in shorter time and was thus chosen for bigger HCC or multiple nodules. However, the cost of the MWA needle was higher compared to RFA (data not shown); this aspect lightly influenced in the first months of the study the choice of thermal ablation type, favoring RFA. These data explain the longer period of follow-up in patients treated with RFA compared to MWA, but did not affect the results on effectiveness of therapy. These are the main reasons for choosing one treatment or the other one. In order to compare data, further analysis was performed on 250 nodules with a diameter of 15 to 35 mm. In this subgroup, the treatment allocation was comparable, and patients were similar in terms of clinical features, number, and ultrasound features of the HCC. Our data showed that MWA was superior to RFA in obtaining a complete necrosis as well as overall survival in 21–35 mm HCC nodules, while no significant difference was observed for smaller nodules. Moreover, overall survival was higher in patients achieving complete necrosis independent of the type of therapy. The results of our study could direct clinicians to choose MWA over RFA in HCC nodes with a diameter of 21 to 35 mm, and this can therefore improve the standard therapeutic approach to HCC therapy.

In conclusion, our study shows that MWA is preferable to RFA in achieving complete response, lower recurrence rate, and higher overall survival rate in HCC nodules with a diameter from 21 to 35 mm: in early, but not in very early HCC, MWA was superior to RFA.

## Figures and Tables

**Table 1:** Features of patients treated with MWA and RFA ablation.

Patients n = 251	MWA n = 81	RFA n = 170	*p* Value
Age, median years (IQR)	67 (57–73)	63 (56–72)	0.584
Sex, M/F (M%)	62/19 (76.5)	118/52 (69.4)	0.294
BMI, median Kg/m^2^ (IQR)	25.2 (22.5–28.1)	25.7 (23.4–28.3)	0.618
Smoke habit, n (%)			
• Ex smokers	32 (39.5)	49 (28.8)	0.262
• Active smokers	25 (30.9)	59 (34.7)	
Alcohol intake, n (%)			
• Ex alcoholic	45 (55.6)	80 (47.1)	0.407
• Active alcoholics	12 (14.8)	27 (15.5)	
Child-Pugh Score, n (%)			
• A	71 (87.7)	148 (87.1)	1.000
• B	10 (12.3)	22 (12.9)	
Albumin, median g/dL (IQR)	4 (3.5–4.3)	3.7 (3.3–4.2)	<0.050
AFP, median ng/mL (IQR)	6.3 (3.6–25.2)	9.5 (4–23)	0.657
Ascites, n (%)	9 (11.1)	22 (12.9)	0.838
Number of patients with 1 or 2 or 3 treated nodules, n (%)			
• 1 nodule	62 (76.5)	127 (74.7)	0.511
• 2 nodules	13 (16)	35 (20.6)	
• 3 nodules	6 (7.4)	8 (4.7)	
Follow up time, median years (IQR)	1.7 (0.9–3.2)	2.9 (1.6–4.3)	<0.001
LT, n (%)	18 (22.2)	36 (21.2)	0.870

MWA = microwave ablation; RFA = radiofrequency ablation; IQR = interquartile range; M = male; F = female; BMI = body mass index; AFP = alfa-fetoprotein; LT = liver transplantation.

**Table 2:** Features of all included HCC nodules (n = 331) treated with thermal ablation.

Nodules n = 331	MWA n = 106	RFA n = 225	*p* Value
Diameter, median mm (IQR)	29 (20–35)	20 (15–25)	<0.001
Diameter			
• ≤14 mm, n (%)	5 (4.7)	48 (21.3)	<0.001
• 15–20 mm, n (%)	22 (20.8)	81 (36)	
• 21–35 mm, n (%)	56 (52.8)	91 (40.4)	
• ≥36 mm, n (%)	23 (21.7)	5 (2.2)	
Infiltrative nodules, n (%)	4 (3.8)	3 (1.3)	0.384
Complex position, n (%)	23 (21.7)	56 (24.9)	0.582
Poor US visibility, n (%)	8 (7.5)	34 (15.1)	0.075

MWA = microwave ablation; RFA = radiofrequency ablation; US = ultrasonography.

## Supplementary Materials

The following are available online at https://www.mdpi.com/1718-7729/28/2/101/s1, Supplementary Table S1. Features of 250 HCC nodules with diameter from 15 to 35 mm.
