# Supplementary material for: Higher Efficiency of Percutaneous Microwave (MWA) Than Radiofrequency Ablation (RFA) in Achieving Complete Response in Cirrhotic Patients with Early Hepatocellular Carcinoma
Source: Curr Oncol. 2021 Feb 25;28(2):1034–44. doi: 10.3390/curroncol28020101 (PMC8025753; doi:10.3390/curroncol28020101)
Supplement: Supplementary file 1 [file curroncol-28-00101-s001.pdf]

| Nodules<br>n=250 | MWA<br>n=78 | RFA<br>n=172 | p value |
|---|---|---|---|
| Diameter, median mm (IQR) | 25 (20-30) | 21 (18-25) | < 0.001 |
| Diameter<br>• 15-20 mm, n (%)<br>• 21-35 mm, n (%) | <br>22 (28.2)<br>56 (71.8) | <br>81 (47.1)<br>91 (52.9) | <br>< 0.05 |
| Infiltrative nodules, n (%) | 2 (2.6) | 3 (1.7) | 0.857 |
| Complex position, n (%) | 19 (24.4) | 42 (24.4) | 1.000 |
| Poor US visibility, n (%) | 6 (7.7) | 19 (11) | 0.5 |
